# The biological inorganic chemistry of zinc ions^☆^

**DOI:** 10.1016/j.abb.2016.04.010

**Published:** 2016-12-01

**Authors:** Artur Krężel, Wolfgang Maret

**Affiliations:** a Laboratory of Chemical Biology, Faculty of Biotechnology, University of Wroclaw, Joliot-Curie 14A, 50-383 Wroclaw, Poland; b King's College London, Metal Metabolism Group, Division of Diabetes and Nutritional Sciences, Department of Biochemistry, Faculty of Life Sciences of Medicine, 150 Stamford Street, London, SE1 9NH, UK

**Keywords:** zinc, zinc ions, zinc biochemistry, zinc biophysics, zinc speciation

## Abstract

The solution and complexation chemistry of zinc ions is the basis for zinc biology. In living organisms, zinc is redox-inert and has only one valence state: Zn(II). Its coordination environment in proteins is limited by oxygen, nitrogen, and sulfur donors from the side chains of a few amino acids. In an estimated 10% of all human proteins, zinc has a catalytic or structural function and remains bound during the lifetime of the protein. However, in other proteins zinc ions bind reversibly with dissociation and association rates commensurate with the requirements in regulation, transport, transfer, sensing, signalling, and storage. In contrast to the extensive knowledge about zinc proteins, the coordination chemistry of the “mobile” zinc ions in these processes, i.e. when not bound to proteins, is virtually unexplored and the mechanisms of ligand exchange are poorly understood. Knowledge of the biological inorganic chemistry of zinc ions is essential for understanding its cellular biology and for designing complexes that deliver zinc to proteins and chelating agents that remove zinc from proteins, for detecting zinc ion species by qualitative and quantitative analysis, and for proper planning and execution of experiments involving zinc ions and nanoparticles such as zinc oxide (ZnO). In most investigations, reference is made to zinc or Zn^2+^ without full appreciation of how biological zinc ions are buffered and how the d-block cation Zn^2+^ differs from s-block cations such as Ca^2+^ with regard to significantly higher affinity for ligands, preference for the donor atoms of ligands, and coordination dynamics. Zinc needs to be tightly controlled. The interaction with low molecular weight ligands such as water and inorganic and organic anions is highly relevant to its biology but in contrast to its coordination in proteins has not been discussed in the biochemical literature. From the discussion in this article, it is becoming evident that zinc ion speciation is important in zinc biochemistry and for biological recognition as a variety of low molecular weight zinc complexes have already been implicated in biological processes, e.g. with ATP, glutathione, citrate, ethylenediaminedisuccinic acid, nicotianamine, or bacillithiol.

## Highlights

• Zinc ions not bound to proteins have critical roles in cell biology.
• Zinc has a unique coordination chemistry, poorly appreciated in the biosciences.
• Its coordination chemistry is significantly different from that of calcium ions.
• Specific conditions apply for buffering cellular zinc ions.
• Investigations with zinc need to consider solution chemistry and metal buffering.

## 1 Introduction

Proteins are the major ligands for zinc(II) ions (“zinc”). Zinc can be readily available from proteins or not available at all unless the protein is degraded. In a way, the coordination chemistry of zinc in proteins bridges the one in natural waters, where zinc is in an available form, and the one in minerals, where zinc is unavailable without chemical processing. Zinc is essential for virtually all cellular functions through its presence in an estimated 3000 human proteins, the zinc sites of which have been predicted by bioinformatics approaches from the signature motifs in their protein sequences [1]. If one considers additional functions of zinc in regulation, the number of zinc proteins in the human zinc proteome will be even larger [2]. The coordination chemistry of zinc in proteins and peptides involves N, O, and S donors of the side chains of histidine, glutamate/aspartate, and/or cysteine with any permutation of these ligands and with the number of protein ligands ranging from three to six. The ligands may not stem from a single protein but from up to four proteins. One property regularly cited for zinc is its flexibility in coordination due to the lack of ligand field stabilization. This allows for dynamic coordination environments of zinc ions, which is critical for example in its catalytic prowess in enzymes when adopting different coordination numbers in interactions with substrates. A critical chemical issue that is important for the functions of proteins is their affinity towards zinc. Using a limited number of ligand donors and geometries, proteins must control and adjust affinities for zinc according to physiological functions. These functions are determined by the structural properties of zinc binding sites such as the presence of a second coordination sphere that engages in hydrogen bonding to ligands, including water molecules when present, geometric strains (entasis), pockets with different dielectric constants (hydrophobic cores), all of which modulate the properties of the bound zinc. For example, in zinc fingers with a ββα fold and in zinc-containing LIM domains, hydrogen bonds and hydrophobic and electrostatic interactions around the bound zinc affect binding and exchange kinetics [3], [4]. Such stabilization through interactions in the second coordination sphere, though not always directly obvious from inspection of the protein structure, occurs in both intra- or intermolecular zinc binding sites and is responsible for stabilizing some sites to affinities for zinc as high as femtomolar in zinc-binding domains such as the zinc hook [5]. A lack or a limited number of stabilizing secondary interactions also can have the opposite effect, namely decreasing affinities of proteins for zinc as a way of controlling their function [6]. Zinc coordination in proteins has been reviewed and catalogued extensively and hence is not the subject of this article. An important subject that has not been reviewed in the literature, however, is the biological coordination chemistry of zinc ions when not bound to proteins – the focus of this article. This subject matter is important with regard to the way zinc is controlled in biology and the functions of zinc as a signalling ion as discussed in many articles in this special issue.

## 2 The relationship between protein-bound and “free zinc” and the role of zinc ions in cellular signalling

Biologically essential divalent metal ions are regulated in cells in ranges of concentrations that follow the Irving-Williams series [7]. If metal ions were not buffered in certain ranges, each metal ion would not be capable of performing its specific functions because the coordination environments of proteins are not sufficiently selective for a particular metal ion. Zinc binds with rather high affinities to metal sites of proteins and therefore is highly competitive towards other metal ions binding with lower affinities. Characteristic for the interaction of zinc ions with intracellular (i) or extracellular (e) proteins (P) is an equilibrium far to the left (Eq. (1)).

(1) $$\text{ZnP}⇄\text{P}+{\left[{\text{Zn}}^{2+}\right]}_{i,e}$$

In order for zinc not to bind to metal sites for iron, for example, the concentrations of zinc ions must be kept sufficiently low despite the fact that total cellular zinc concentrations are rather high, ie. about 200 μM. This control is achieved by buffering zinc in a range of relatively low pZn (−log[Zn^2+^]_free_), a situation quite different from solutions of zinc salts in water or in most biological buffers where zinc is not buffered. The term “zinc buffering” is based on the pH buffering concept, where an appropriate pH value is controlled by the ratio between a protonated form (HA), formally an acid according to the Brønsted–Lowry theory, and a deprotonated form of the buffer component (A^−^), formally a base, and the acid dissociation constant of HA (p*K*_a_) (Eq. (2), the Henderson–Hasselbalch equation). Likewise zinc buffering and pZn are determined by both species of the zinc buffering components, ie. the ratio between the metallated holo-form of the zinc complex ZnP (if the ligand is a protein, P) and the demetallated apo-form P, and the dissociation constant (p*K*_d_) of the ZnP complex (Eq. (3)) [8]. Zinc buffering must occur in a way to have most zinc enzymes 100% metallated to be functional. In cultured HT29 colon cancer cells, about 10% of sites with high affinity (*K*_d_ = 83 pM) were found to be demetallated, thus contributing to a ∼10/90% buffer ratio under these conditions [9].

(2) $$\text{pH}=\text{p}K_{\text{a}}+\text{log}\left(\frac{\left[{\text{A}}^{-}\right]}{\left[\text{HA}\right]}\right)$$

(3) $$\text{pZn}=\text{p}K_{\text{d}}+\text{log}\left(\frac{\left[\text{P}\right]}{\left[\text{ZnP}\right]}\right)$$

The concentrations of “free” zinc ions in the cytosol of cells have been estimated to be in the range of a few hundred picomolar under steady-state conditions [9], [10]. They can fluctuate only in a rather narrow range to avoid deficiency and toxicity. The efficient zinc buffering is achieved by high affinity binding to proteins and transport processes that also control the concentrations of zinc. In order to allow for and control pre-steady state fluctuations of zinc ions, an additional process participates, namely the transport of excess zinc ions into a subcellular store. This kinetic process contributes to thermodynamic buffering and is called muffling [11]. A muffler binds zinc ions and transports them from the cytosol to a vesicular compartment where they are sequestered. Muffling allows the cell to cope with higher zinc ion fluctuations without the need for high buffering capacity and makes possible zinc ion fluxes and signalling in an otherwise metal-buffered environment. Buffering and muffling have a limited capacity. Yet, they avoid that zinc ions reach higher concentrations to displace other metal ions from their binding sites or to bind to target sites that are normally not occupied with metal ions.

On the other hand, sufficient zinc ions must be available to supply zinc-requiring proteins. Zinc is released during protein turnover, transported into the cell and out of the cell, and further re-distributed among cellular compartments. The control of total and “free” zinc ion concentrations also involves fluctuating zinc ion concentrations that regulate biological processes. Increased cellular zinc ion concentrations (“zinc ion transients” or “zinc signals”) are generated during mobilization of zinc ions from proteins by chemical processes that modify the protein or release of zinc ions from organellar/vesicular stores, where zinc is stored in a yet to be determined chemical form (Eq. (4)).

(4) $$\text{ZnP}\to \text{P}*+{\left[{\text{Zn}}^{2+}\right]}_{i,e}\;\text{where}\;\text{P*}\;\text{denotes}\;\text{a}\;\text{modified}\;\text{protein}$$

Similar to the release of calcium ions, hormones were shown to stimulate the release of zinc ions from the endoplasmic reticulum (ER) [12]. However, an examination of epidermal growth factor/ionomycin stimulated breast cancer cells employing genetically encoded FRET sensors for zinc could not detect the release of zinc ions [13]. Signalling with oxidative species also can release zinc ions from coordination environments of zinc with sulfur donors from cysteine in proteins, linking redox metabolism and zinc metabolism and converting redox signals into zinc signals [14]. While the hydrated Ca^2+^ ion is the major species in calcium signalling, this is not the case in zinc signalling as the Zn^2+^ ion has much higher affinities for donors of ligands.

Furthermore, some cells release zinc ions and increase extracellular zinc concentrations. The pool of zinc ions that is not bound to proteins is functionally significant and has been variously referred to as “labile zinc”, “free zinc”, “fast-exchanging zinc”, and “mobile zinc.” All these operational definitions are unsatisfactory as chemical terms. The use of these terms has resulted in considerable ambiguities. In the entire biochemical literature, the zinc ions not bound to proteins are not specified in terms of their coordination environments. In analogy with Ca^2+^ in biology, some investigators think of “free” zinc as its aquo complex. The chemist understands “free” zinc as the ion without ligands, which exists only in the gas phase. However, given the large number of potential ligands inside and outside cells, and the dynamic changes of ligands and zinc concentrations, non-protein bound (“free”) zinc is expected to have different types of LMW ligands under various biological conditions, including inorganic and organic anions, and in this regard it is quite different from Ca^2+^. The lack of understanding the chemical nature of the pool of non-protein bound zinc is a major limitation in interpreting experiments and in planning experiments with conclusive outcomes. In this article, we attempt to address this issue by discussing the solution chemistry of zinc ions and then relating it to the biological coordination chemistry, with an emphasis on quantitative concepts and the constraints imposed by cells. This treatment results in a focus on those aspects of zinc chemistry that are important for biology, nutrition, pharmacology and toxicology, and for investigating the biology of zinc with fluorescent probes. The characteristic coordination chemistry of zinc is a key to understanding its physiological and pathophysiological functions in living organisms.

## 3 Zinc speciation

**Fig. 1:**
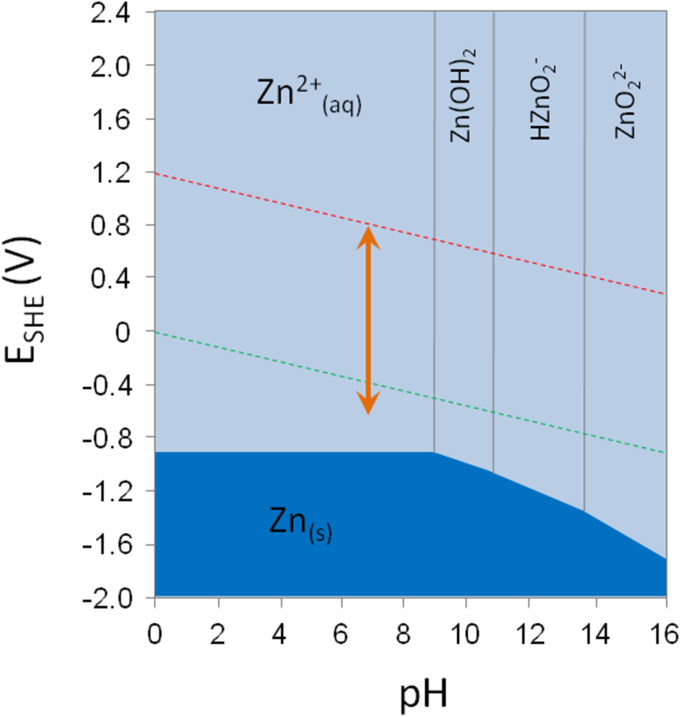
Pourbaix diagram for the speciation of zinc. Red and green dashed lines demonstrate two possible cathodic reactions, oxygen reduction (oxygen dissolved in water in equilibrium with water) and hydrogen ion reduction (water in equilibrium with gaseous hydrogen), respectively. The orange arrow shows the range of biological standard reduction potentials at pH 7.0: from ∼820 mV to ∼ −670 mV.

The speciation of zinc is illustrated best with a Pourbaix diagram (Fig. 1). Such a diagram shows chemical species as a function of both redox standard potential and pH values, and demonstrates a remarkable property of zinc that is critical for its functions in biology: *In the absence of other coordinating ligands*, zinc is present as hydrated Zn^2+^_(aq)_, over the entire ranges of redox potentials and pH values in biology. The orange arrow in Fig. 1 indicates the range of biologically important standard potentials determined at pH 7.4. Even for the lowest half reactions of the reductions of acetate to acetaldehyde (−581 mV) or succinate to α-ketoglutarate (−670 mV) zinc maintains its the +2 oxidation state [15]. In other words, zinc is redox-inert in biology and therefore its redox properties are irrelevant. Therefore, we refer to zinc in the +2 oxidation state (Zn(II)) simply to zinc in this article though this term is reserved to the element in the 0 oxidation state in chemistry. In biology, many ligands are present and the species observed as a function of the concentrations of these ligands and pH are relevant as they modify the behavior of zinc ions. The resulting implications for function are discussed in this article.

### 3.1 Zinc(II) aquo complexes and hydroxides

The Pourbaix diagram (Fig. 1) does not imply that hydrated Zn^2+^_(aq)_ is a single species. The chemistry of the zinc ion in water is fundamental, rather complex, and has important consequences for the role of zinc in biology. Water is both a Lewis base, coordinating as a ligand via its oxygen atom to the zinc ion, and a Lewis acid, coordinating to donors with negative charges via its hydrogen atoms. Binding of water molecules is electrostatic in nature for alkali metal ions, such as Na^+^ and K^+^, but has significant covalent character for transition metal ions including zinc ions. The covalent character of the zinc ion/water interaction is confirmed by a high heat of zinc ion solvation of ∼ −500 kcal/mol in aqueous solutions [16]. Assuming six water molecules (hexaaquo complex) bound to a central zinc ion, the enthalpy of solvation for one water molecule is ∼ −80 kcal/mol, which corresponds to typical values for covalent Zn-O bond formation (−70 to −85 kcal/mol).

Zn^2+^ has a filled d-shell ([Ar]d^10^ electron configuration) and consequently no ligand field stabilization energies and associated stereochemical preferences. Therefore, coordination number and geometry of zinc complexes are determined mainly by the radii of both the metal ion and the coordinating ligand atoms or ions and its aquo complexes have quite flexible coordination geometry. Besides the commonly described hexaaquo complex [Zn(H_2_O)_6_]^2+^, there is also a tetraaquo complex [Zn(H_2_O)_4_]^2+^. In the solid state, a pentacoordinate [Zn(H_2_O)_5_]^2+^ complex has also been found [17]. *Ab initio* calculations show that hexa-, penta- and tetraaquo zinc complexes differ by less than 0.4 kcal/mol, which is in contrast with other divalent cations such as magnesium. It is in agreement with the biological roles of the cations. The coordination of magnesium is mostly octahedral whereas zinc ions are bound to sulfur, nitrogen or oxygen donors with coordination dynamics linked to catalysis [18].

Zinc aquo complexes are acids according to the Brønsted*–*Lowry theory. The polarization of water molecules by the central zinc ion causes dissociation of a proton, resulting in the formation of an aquo-hydroxo complex (Eq. (5)). The equation solved for the concentrations of protons [H_3_O^+^] is the non-logarithmic form of the Henderson-Hasselbalch equation describing pH buffering of a zinc salt in the range of pH within ±1 of the p*K*_a_ (p*K*_a1_ = 9.05) of hydrolysis of the hexaaquo complex [19] (Eq. (6)).

(5) $${\left[\text{Zn}{\left({\text{H}}_{2}\text{O}\right)}_{6}\right]}^{2+}+{\text{H}}_{2}\text{O}⇄{\left[\text{Zn}\left(\text{OH}\right){\left({\text{H}}_{2}\text{O}\right)}_{5}\right]}^{+}+{\text{H}}_{3}{\text{O}}^{+}$$

(6) $${\left[{\text{H}}_{3}\text{O}\right]}^{+}=K_{\text{a}1}\frac{{\left[\text{Z}\text{n}{\left({\text{H}}_{2}\text{O}\right)}_{6}\right]}^{2+}}{{\left[\text{Z}\text{n}\left(\text{O}\text{H}\right){\left({\text{H}}_{2}\text{O}\right)}_{5}\right]}^{+}}$$

All zinc salts of strong acids (chloride, nitrate, sulfate or perchlorate) are weak acids as indeed observed in pH-metric measurements. The pH value of a solution of a zinc salt in water can be calculated from Eq. (7), which is derived from Eq. (6) under the assumptions that the concentrations of H_3_O^+^ and [Zn(OH)(H_2_O)_5_]^+^ are the same and the concentration of [Zn(H_2_O)_6_]^2+^ corresponds roughly to the total concentration (c_s_) of the zinc salt.

(7) $$\text{pH}=0.5\text{p}K_{a1}-0.5\;\text{log}\left(c_{s}\right)$$

Based on Eq. (7) a 0.05 M solution of a zinc salt in water has a pH of 5.18. The higher the zinc salt concentration the lower is the pH value. This pH value refers to solution of a zinc salt with an undefined anion. In practice, the pH of solutions of zinc salts depends not only on concentrations but also on the chemical composition of the salt. Solutions of zinc salts therefore can be acidic, neutral or even slightly basic with pH = 7 (neutral) as the relative reference point on the acid-base scale. For example, the pH values of 1–10% (w/w) ZnSO_4_⋅7H_2_O (0.035–0.35 M) or ZnCl_2_ (0.074–0.74 M) solutions are between 4.4 and 5.9 depending on the commercial source of the salt. These pH values are in good agreement with calculations obtained from Eq. (7). However, the pH values of 1–10% Zn(OAc)_2_ (0.05–0.55 M) solutions are between 6.0 and 8.0, which is significantly different from those of the above mentioned salts. The origin of the difference in pH values is the acid-base properties of the anion. Because sulfate and chloride are anions of strong acids, they are extremely weak bases and do not affect the pH of the aqueous solution. The reason for the different behavior observed for zinc acetate, citrate, or formate solutions is that these anions have basic character and hence oppose the effects on pH due to [Zn(H_2_O)_6_]^2+^ hydrolysis. Acetate reacts with water according to Eq. (8) and decreases the pH.

(8) $${\text{CH}}_{3}{\text{COO}}^{-}+{\text{H}}_{2}\text{O}⇄{\text{CH}}_{3}{\text{COOH}+\text{OH}}^{-}$$

The p*K*_b_ (= p*K*_w_ - p*K*_a_) value of the acetate/acetic acid pair is 9.2 and similar to the p*K*_a1_ of [Zn(H_2_O)_6_]^2+^ (Eq. (6)). Because these opposing effects cancel each other, the pH values of Zn(OAc)_2_·*n*H_2_O solutions are neutral despite the fact that cation and anion have acid-base properties. For practical purposes, it is important to note that the pH value of zinc salts varies slightly depending on manufacturers and impurities, the carbon dioxide concentration, and the time of storage. It is also useful to remember that zinc salts with anions of strong acids have low pH-buffering capacity. Therefore, addition of a pH buffer at physiological pH to a solution of ZnCl_2_ will immediately change the pH of the solution, possibly resulting in the precipitation of zinc hydroxide. On the other hand, a Zn(OAc)_2_ solution is a better pH-buffer. Its pH-buffering capacity depends on its concentration and therefore preparation of too concentrated solutions also may result in a pH that causes precipitation of zinc hydroxide. In summary, zinc salt solutions in biological buffers without capacity for zinc complexation may provide a rather poorly controlled source of zinc ions for biological experiments.

Amphoteric characteristics of zinc aquo complexes are evident from further deprotonation of water molecules and formation of hydroxo species. The octahedral zinc hexaaquo complex [Zn(H_2_O)_6_]^2+^ is in equilibrium with tetrahedral [Zn(H_2_O)_4_]^2+^ with an equilibrium constant much in favor of the former. For simplicity, the equilibria of aquo complexes with various geometry, [Zn(H_2_O)_*x*_]^2+^ will refer to either hexaaquo (*x* = 6) or tetraaquo (*x* = 4) complexes in this article. Vibrational spectroscopic investigations on aqueous solutions of zinc salts demonstrated formation of the tetraaquo complex at high salt concentrations where the water content decreases significantly [20]. The zinc pentaaquo-hydroxo complex also is in equilibrium with the octahedral and tetrahedral species. The conversion of [Zn(OH)(H_2_O)_5_]^+^ to [Zn(OH)(H_2_O)_3_]^+^ has not been described in detail in terms of its equilibrium constant/energetics. The dissociation of another water molecule results in the formation of a neutral [Zn(OH)_2_(H_2_O)_*x-2*_]_(aq)_ species. The octahedral species [Zn(OH)_2_(H_2_O)_4_]_(aq)_ is less favored due to additional charge compensation and stronger repulsion of negatively charged ligands, all of which favor the tetrahedral [Zn(OH)_2_(H_2_O)_2_]_(aq)_ species. *Ab initio* calculations show that the p*K*_a_ value for the dissociation of a proton from a water molecule bound to an octahedral zinc complex is about 0.8 logarithmic units higher compared to that from a water molecule bound to a tetrahedral zinc complex [21]. This phenomenon is important for enzyme catalysis, where a tetrahedral zinc(II) provides a significant advantage in terms of providing a higher fraction of the hydroxo species as a nucleophile at physiological pH. The chemistry of aquo and aquo-hydroxo species of zinc is quite different from calcium and in addition to functions in proteins may be important in biological recognition of zinc. Macromolecules sometimes select a minor species from an assembly of species as a ligand or substrate.

Due to our limited knowledge regarding equilibria between octahedral and tetrahedral zinc aquo-hydroxo complexes, these complexes are frequently presented in a simplified way as Zn(OH)^+^, Zn(OH)_2(aq)_, Zn(OH)_3_^-^ and Zn(OH)_4_^2−^ which is a simplification focusing only on the number of hydroxo ligands. The stepwise proton dissociation constants of the second (p*K*_a2_ = 9.75), third (p*K*_a3_ = 10.1) and fourth (p*K*_a4_ = 10.5) water molecule bound to the central zinc ion are average values of the dissociation constants of both octahedral and tetrahedral species, the molar fraction of which changes with pH [19]. Eqs. (9), (10), (11) present stepwise dissociations of all zinc aquo-hydroxo complexes. The final product, the [Zn(OH)_4_]^2-^ complex (zincate), is tetrahedral and has no covalently bound water molecules because of its high negative charge. This charge effect forces octahedral complexes present at acidic and neutral pH into a tetrahedral complexes at alkaline pH.

(9) $${\left[\text{Zn}\left(\text{OH}\right){\left({\text{H}}_{2}\text{O}\right)}_{x-1}\right]}^{+}+{\text{H}}_{2}\text{O}⇄{\left[\text{Zn}{\left(\text{OH}\right)}_{2}{\left({\text{H}}_{2}\text{O}\right)}_{x-2}\right]}_{\left(\text{aq}\right)}+{\text{H}}_{3}{\text{O}}^{+}$$

(10) $${\left[\text{Zn}{\left(\text{OH}\right)}_{2}{\left({\text{H}}_{2}\text{O}\right)}_{x-2}\right]}_{\left(\text{aq}\right)}+{\text{H}}_{2}\text{O}⇄{\left[\text{Zn}{\left(\text{OH}\right)}_{3}{\left({\text{H}}_{2}\text{O}\right)}_{x-3}\right]}^{-}+{\text{H}}_{3}{\text{O}}^{+}$$

(11) $${\left[\text{Zn}{\left(\text{OH}\right)}_{3}{\left({\text{H}}_{2}\text{O}\right)}_{x-3}\right]}^{-}+{\text{H}}_{2}\text{O}⇄{\left[\text{Zn}{\left(\text{OH}\right)}_{4}\right]}^{2-}+{\text{H}}_{3}{\text{O}}^{+}$$

where *x* = 4 or 6 depending on complex geometry.

**Fig. 2:**
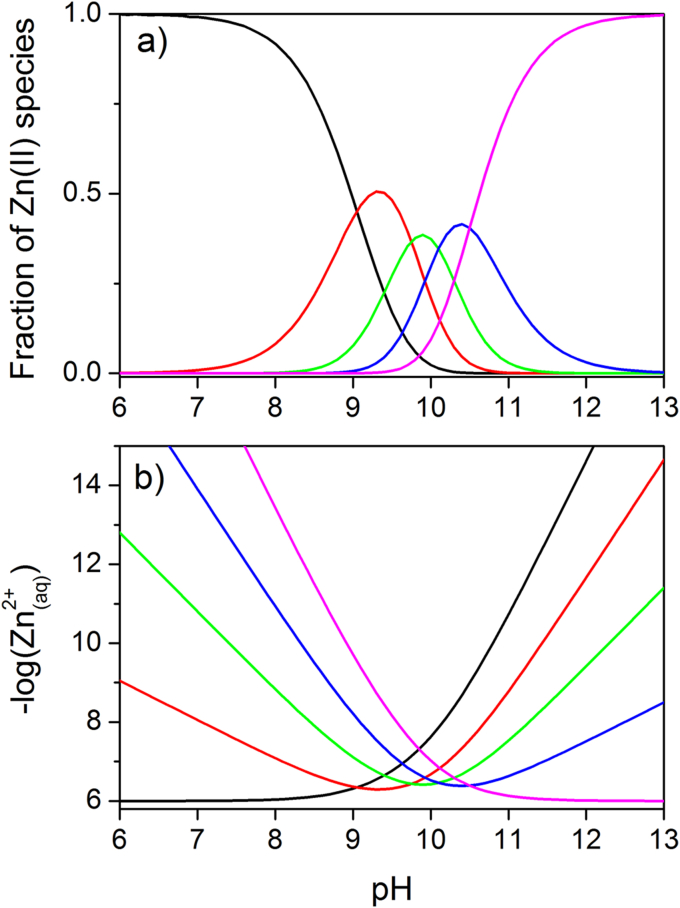
The pH dependence of five zinc aqua-hydroxo complexes in water solution. a) Molar fraction distribution of particular zinc species as a function of pH. b) Logarithmic plot of the concentration of particular species [17]. Black, red, green, blue and magenta color lines correspond to [Zn(H_2_O)_*x*_]^2+^, [Zn(OH)(H_2_O)_*x-1*_]^+^, [Zn(OH)_2_(H_2_O)_*x-2*_]_(aq)_, [Zn(OH)_3_(H_2_O)_*x-3*_]^-^, and [Zn(OH)_4_]^2-^, respectively.

The pH dependence of the aquo-hydroxo complexes is based on these equilibria (Fig. 2). The deprotonation of coordinated water molecules begins at slightly alkaline pH, above 7.8 (5% of [Zn(OH)(H_2_O)_*x-1*_]^+^), and terminates with the fully deprotonated species above pH 11.8 (95% of [Zn(OH)_4_]^2-^). The concentration of the uncharged complex, [Zn(OH)_2_(H_2_O)_*x-2*_]_(aq)_, is highest at pH 9.9 and decreases above that value.

Solid ZnO is formed by calcination (heating at high temperatures) from Zn(OH)_2(s)_ and widely used in nutritional and some research applications. A discussion of the properties of Zn(OH)_2(s)_ and ZnO illustrates the issues associated with the dissolution of different forms of these compounds and hence availability of zinc for biological systems. Precipitation of solid zinc hydroxide Zn(OH)_2(s)_ observed in mildly alkaline aqueous solutions is strictly related to the presence of the uncharged, but water soluble [Zn(OH)_2_(H_2_O)_*x-2*_]_(aq)_ species. Transition from the soluble to the insoluble form requires the dissociation of water. The type of Zn(OH)_2(s)_ crystals formed depend on the total zinc concentration, pH, and temperature. Six forms of Zn(OH)_2(s)_ have been described so far, and they are known as α (amorphous) and crystalline β_1_, β_2_, γ, δ and ε. They have widely variable solubility in water according to the series: amorphous > β_1_ ∼ γ > β_2_ > ε [22]. Another confounding variable in the determination of the solubility is the use of ZnO. In this case, the solubility and thermodynamic equilibration of hydroxo complexes is influenced by the slow hydration kinetics of ZnO to yield Zn(OH)_2(s)_ and soluble species of the type [Zn(OH)_2_(H_2_O)_*x-2*_]_(aq)_. Furthermore, ε-Zn(OH)_2(s)_ is unstable with respect to ZnO_(s)_ and H_2_O and also with respect to amorphous Zn(OH)_2(s)_, and the conversion between the various forms of Zn(OH)_2(s)_ and ZnO is often slow [22]. ZnO has lower solubility compared to the amorphous form of Zn(OH)_2(s)_ (one order of magnitude difference in *K*_SO_). Clear differences in the kinetics of transition between ZnO and different forms of Zn(OH)_2_ and in their solubilities are the reason why different procedures result in the production of different forms of ZnO.

Best characterized is the solubility of the orthorhombic form ε-Zn(OH)_2(s)_ with a *K*_SO_ of 3.39 × 10^−17^ (p*K*_SO_ = 16.47) at 25 °C and *I* = 0.2 M [22]. Based on the solubility product (*K*_SO_ = [Zn^2+^_(aq)_] [OH^−^]^2^) and the ionic product of water *K*_w_ one can readily calculate the free zinc ion concentration or the pH of a solution of ε-Zn(OH)_2_ according to Eq. (12).

(12) $$\left[{\text{Zn}}_{\left(\text{aq}\right)}^{2+}\right]=\frac{K_{\text{SO}}}{{\left[{\text{OH}}^{-}\right]}^{2}}=\frac{K_{\text{SO}}{\left[{\text{H}}^{+}\right]}^{2}}{K_{\text{w}}^{2}}$$

When calculating the solubility of pH-sensitive solids such as Zn(OH)_2(s)_, it is important to include the effect of hydroxo complex formation as a factor that increases the total solubility of Zn(OH)_2(s)_ (Fig. 2). Thus, solubility (S) is the sum of all the soluble species discussed above at a particular pH value (Eq. (13)).

(13) $$\text{S}={\left[\text{Zn}{\left({\text{H}}_{2}\text{O}\right)}_{x}\right]}^{2+}+{\left[\text{Zn}\left(\text{OH}\right){\left({\text{H}}_{2}\text{O}\right)}_{x-1}\right]}^{+}+{\left[\text{Zn}{\left(\text{OH}\right)}_{2}{\left({\text{H}}_{2}\text{O}\right)}_{x-2}\right]}_{\left(aq\right)}+{\left[\text{Zn}{\left(\text{OH}\right)}_{3}^{-}{\left({\text{H}}_{2}\text{O}\right)}_{x-3}\right]}^{-}+{\left[\text{Zn}{\left(\text{OH}\right)}_{4}\right]}^{2-}\text{where}\;x=4\;\text{or}\;6$$

Concentrations of a particular species can be calculated based on the individual dissociation constants for each step. However cumulative formation constants (*β*_1-4_) are much more convenient in practice in order to simplify the computation. According to this notation *β*_1_ (*K*_1_), *β*_2_ (*K*_1_ × *K*_2_) … are [Zn(OH)^+^]/[Zn^2+^][OH^−^], [Zn(OH)_2(aq)_]/[Zn^2+^][OH^−^]^2^ … and their logarithmic values are 4.95 (*β*_1_), 9.20 (*β*_2_), 13.1 (*β*_3_) and 16.6 (*β*_4_). Since the effect of different numbers of coordinated water molecules on these constants is not known, water molecules are omitted here for clarity. When combining Eq. (13) with the concentrations of particular zinc species, the solubility of Zn(OH)_2(s)_ is expressed by Eq. (14). Combining the solubility and solubility constants (*K*_SO_) allows for calculation of the total concentrations (sum of all Zn(II) forms, Zn^2+^_(aq)_) of soluble species at any given pH (Eq. (15)).

(14) $$\text{S}=\left[{\text{Zn}}_{\left(\text{aq}\right)}^{2+}\right](1+\beta_{1}\left[{\text{OH}}^{-}\right]+\beta_{2}{\left[{\text{OH}}^{-}\right]}^{2}+\beta_{3}{\left[{\text{OH}}^{-}\right]}^{3}+\beta_{4}{\left[{\text{OH}}^{-}\right]}^{4}$$

(15) $$\text{S}=K_{\text{SO}}\left(\frac{1}{{\left[{\text{OH}}^{-}\right]}^{2}}+\frac{\beta_{1}}{\left[{\text{OH}}^{-}\right]}+\beta_{2}+\beta_{3}\left[{\text{OH}}^{-}\right]+\beta_{4}{\left[{\text{OH}}^{-}\right]}^{2}\right)$$

**Fig. 3:**
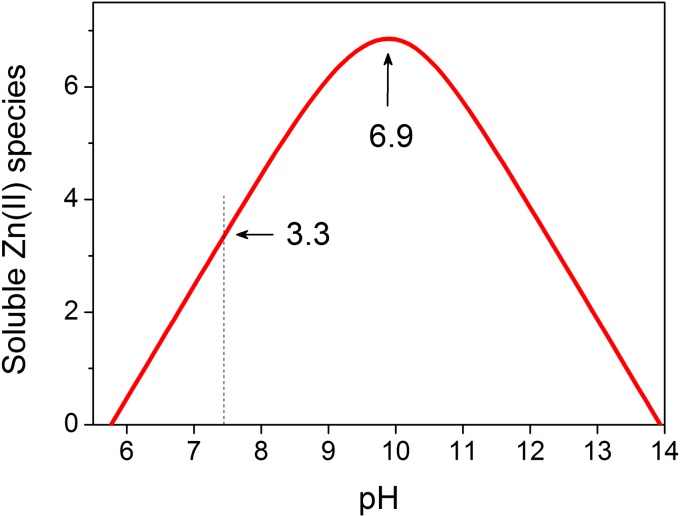
The pH dependence of the solubility of zinc hydroxide (ε-Zn(OH)_2(s)_) [20]. The ordinate indicates on a logarithmic scale the sum of all zinc soluble species present at a particular pH (Zn^2+^_(aq)_) which include aquo- and hydroxocomplexes presented in Fig. 2. The dashed line indicates the −log[Zn^2+^_(aq)_] value at pH 7.4.

Eq. (15) also allows for calculating the solubility of Zn(OH)_2(s)_, which is frequently considered as a completely insoluble zinc species. In a presentation of the relationship between pH and the solubility of zinc hydroxide (Fig. 3), the ordinate gives the sum of all soluble zinc species [Zn^2+^_(aq)_] present at various molar fractions at a particular pH. For example, −log values of the concentration of all soluble Zn(II) species at pH 7 and 7.4 are 2.47 and 3.26, corresponding to 3.4 and 0.55 mM, respectively. Even at pH ∼10, where the solubility of Zn(OH)_2(s)_ is lowest, the concentration of soluble zinc species is 0.13 μM. These values are much higher than what researchers usually estimate. They have been confirmed experimentally, for example by using atomic absorption spectrophotometry [23]. A relatively high solubility of Zn(OH)_2(s)_ is the reason why addition of ZnSO_4_ or ZnCl_2_ at submillimolar concentrations to a HEPES buffer (at a pH close to the p*K*_a_ of the buffer) does not result in precipitation. This fact turns out to be useful when preparing solutions of these zinc salts for biophysical measurements [24].

Another important issue in zinc hydroxo complex chemistry is olation, the formation of polynuclear species, such as [Zn_2_(OH)_6_]^2-^ and complexes of higher nuclearity. Polynuclear complexes are formed rather slowly and many of them are kinetic intermediates in the slow transition from soluble hydroxo mononuclear complexes to a solid precipitate and are thus thermodynamically unstable. The formation of precipitates may be observed when storing stock solutions of zinc salts. Their “aging” has many reasons, such as formation of carbonates or mixed complexes, presence of impurities, and it may also involve the formation of polynuclear species, which are less soluble in water. Upon dilution, the fraction of polynuclear zinc hydroxo complexes in aqueous solutions decreases.

While this presentation of the chemistry of zinc aquo complexes may seem quite involved, nevertheless it is fundamental to zinc in biology and to the preparation of various solutions of zinc salts and complexes for controlled and well-designed experiments in the laboratory and for the feeding of organisms.

Zinc aquo complexes have to exchange their water molecules when binding to other ligands. Binding to any protein site requires such ligand exchange. It is particularly relevant when zinc is transported as transport involes multiple ligand exchanges. The 3D structure of the bacterial zinc transporter *E. coli* YiiP, which belongs to the family of cation diffusion facilitators (CDF), shows that the transported species apparently is a Zn^2+^ ion without bound water molecules or LMW ligands when it is bound in the primary site to one histidine and three aspartate side chains [25]. If a zinc ion without ligands in transfer or transport in a protein exists transiently, we suggest referring to it as a “naked” zinc ion to distinguish it from the “free” zinc ions with bound ligands in solution. The energetics of ligand exchange therefore are critical to the functioning of this and other zinc transporters. The properties of the Zn-O bond in zinc bound-water molecules are at the center of mechanisms of zinc metalloenzymes, in particular the modulation of the p*K*_a_ of the Zn^2+^−OH_2_ to Zn^2+^−OH^−^ transition, which provides the nucleophile for hydrolytic reactions.

Deprotonation of zinc aquo complexes leads to species with different charges and they are important for interaction with proteins. The pH dependent species of zinc aquo complexes are also important when performing experiments with zinc compounds. The acid-base properties need to be considered when making stock solutions of zinc salts or zinc complexes. The type of salt and its concentrations are important with regard to whether or not enough pH buffering capacity is available to keep zinc ions in solution. When stock solutions of zinc salts in water or buffer are added to culture media for cells or bacteria, the solubility and the possible complexation by pH buffers and other components of the media must be understood and considered.

Zinc compounds are used in nutritional studies of organisms. ZnO, for example, has been used in the large age-related eye disease study (AREDS) [26]. When using this compound, it is important to know the chemical properties of the different commercially available products in the corresponding biological environment in order to understand bioavailability. The physical properties, such as solubility and particle size, can vary significantly for ZnO from different suppliers. This variation also applies to manufactured ZnO nanoparticles, which are now being increasingly employed in feeding and investigated with regard to their effects on biological systems.

One issue not addressed above is that a precipitate can be formed when dissolving ZnCl_2_ in water. Despite the high solubility of the salt, the precipitate is due to the formation of zinc oxychlorides, of which at least 16 different forms have been described [27]. One important form is tetrabasic zinc chloride (TBZC), zinc chloride hydroxide monohydrate with the chemical formula Zn_5_(OH)_8_Cl_2_⋅H_2_O [28]. It has a variety of applications in animal feed, as a fungicidal additive to plant food and in medicine/dentistry.

### 3.2 Biologically important anions

In natural waters, zinc speciation has been investigated widely and Zn^2+^_(aq)_, ZnCO_3_^o^, ZnSO_4_^o^, Zn(OH)_2_°, ZnCl^+^, ZnCl_2_, ZnCl_3_^−^, and ZnCl_4_^2−^ have been identified [29]. In this literature, the supscript (o) refers to the soluble forms, ie. Zn(OH)_2_° = Zn(OH)_2(aq)_. The chemical formulae do not indicate the bound water molecules. Thus, ZnCl^+^ is actually [ZnCl(H_2_O)_*x-1*_]^+^ according to the speciation and nomenclature discussed above. The concentrations of these species depend on the pH of the aqueous solution, the anion concentrations and the presence of dissolved organic matter (DOM), which on average contributes with a log*K* of 6.4–7.0 with ligands such as humic and fulvic acid. Chloride species become important with increasing salinity. The concentrations of zinc hydroxo and carbonato complexes increase above pH 7.5 and become the predominant species above pH 8. Sulfide also contributes, even in oxic water. In anoxic water, ZnS nanoparticles and Zn-S clusters have been identified: [Zn_3_S_3_(H_2_O)_6_] and [Zn_4_S_6_(H_2_O)_4_]^4-^ [30], [31]. It is quite remarkable that the nuclearity of these complexes with regard to zinc is exactly what is found in the two zinc-sulfur clusters of mammalian metallothioneins.

**Fig. 4:**
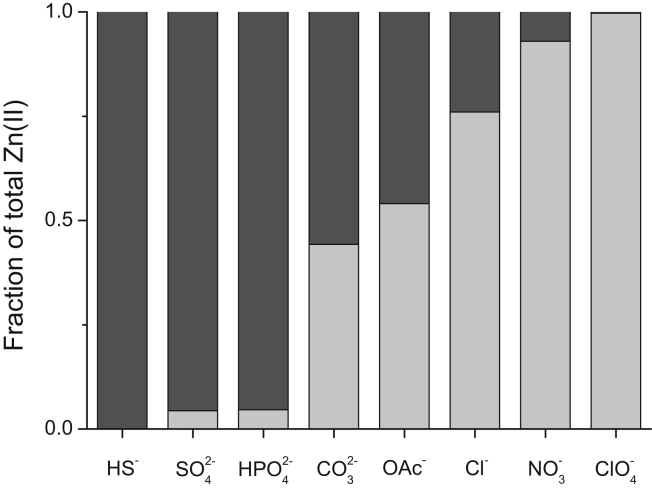
The zinc binding abilities of biological anions. The bars demonstrate percentages of free and complexed species, calculated for 0.1 M anion and 1 μM of total Zn^2+^. Light and dark grey colors refer to free and bound Zn(II) species, respectively.

Distribution of elements in blood often reflects their distribution in sea water. However, within cells, a different milieu is maintained as the concentrations of chloride and hydrogen sulfide (HS^−^) are kept relatively low, though the latter is a signalling substance and a component of the structure of Fe-S clusters. Whether these anions (Table 1) are involved in coordinating zinc ions or the presumable coordination changes during zinc transport from/into cells is not known. In the cell, these anions are also buffered, ie. there is a controlled equilibrium between free and bound, though the term buffering is not used in this context. Hydrogen sulfide is kept at very low concentrations, but hydrogen phosphate (HPO_4_^2−^), sulfate (SO_4_^2−^), hydrogen carbonate/carbonate (HCO_3_^−^), and chloride are all present at millimolar concentrations (Table 1). Hydrogen sulfide, hydrogen phosphate, and sulfate are the strongest inorganic anions for zinc (Fig. 4). There is also diphosphate (pyrophosphate, P_2_O_7_^4−^), triphosphate (P_3_O_10_^5−^), tetraphosphate (P_4_O_13_^6−^), and inositol phosphate (section 4, Table 2), all of which bind zinc much more efficiently with apparent dissociation constants (p*K*_d_) of 2.7, 6.9, 7.2 and 10.4, respectively [32], [33], [34], [35]. Acetate, carbonate, and chloride are ligands with intermediate strength for coordinating with zinc ions, and some anions are biological ligands of zinc in proteins. Free acetate is expected to be very low as it is mainly in the form of acetyl-CoA. Other organic acids that are metabolites can also serve as potential ligands (Table 2). Hydrogen carbonate is a ligand in the zinc enzyme carbonic anhydrase. Chloride has been identified as a ligand of zinc in the crystal structures of some zinc proteins. Chloride is likely to be a ligand in ternary complexes with other ligands if expansion of the coordination sphere is possible. Hydrogen phosphate interacts with zinc sites in metallophosphatases. Chloride complexes are likely significant in the stomach, which contains up to 0.1 M HCl, whereas carbonate complexes likely play a role in the duodenum where Brunner's glands provide an alkaline secretion high in bicarbonate to neutralize the acid from the stomach.

When preparing zinc salt solutions for biological experiments the possibility of these anions forming zinc complexes and affecting the outcome of the experiment(s) is significant and should be considered, in particular in the absence of other ligands with competing affinity for zinc. Physiological salt solutions are often based on phosphates and so are cell culture media, which provide ligands for zinc in the absence of serum. Considering speciation of complexes with anions is not only important for bioavailability of zinc ions but also for interactions with proteins, which may be very specific and lead to either activation or inactivation of a biological process. Sodium or potassium chloride added to biological buffers to adjust their ionic strength will change zinc speciation, especially in the absence of other ligands with high affinity for zinc. Perchlorate does not occur naturally in biological systems and nitrate concentrations are very low (Table 1). Other negatively charged metal anion or oxoanion complexes may also form insoluble zinc complexes. Examples are [Fe(CN)_6_]^4-^ or [Hg(SCN)_4_]^2-^ used in analytical chemistry to precipitate zinc ions.

## 4 The complexation of zinc with ligands other than water and the stability of zinc complexes

All biologically important molecules such as amino acids, peptides, proteins, carbohydrates, nucleotides, DNA and RNA, and vitamins contain atoms that can serve as electron pair donors for metal coordination and therefore can be considered as potential ligands for metal ion binding [36]. However, many interactions may not be strong enough to be physiologically significant. Accordingly, the biological zinc chemistry has focused almost exclusively on proteins.

Ligands are called monodentate if they possess only one donor atom or donate just one of their available pairs of electrons to the metal ion. They are called multidentate if they possess more than two donor atoms. Multidentate ligands can form chelates, ring structures of covalently linked donors around the central metal ion. The highest stability is attained when 5- or 6-membered chelate rings are formed. The prototype of a ligand that forms a 5-membered chelate ring and occupies six coordination positions around the zinc ion is ethylendiaminetetraacetic acid (EDTA). The chelate effect makes an important contribution to enhancing the stability of low molecular weight zinc complexes when compared to monodendate complexes as it increases the entropy of the system [37].

How stable zinc complexes are is determined by the (Gibbs) free energy, which has an enthalpic and an entropic contribution. Changes of either one affect complex stability (Eqs. (16), (17))).

(16) $$\Delta G^{\circ }=-\text{RT}\;\ln\;K=\Delta H^{\circ }-\text{T}\Delta S^{\circ }\text{;}$$

(17) $$\ln\;K=\frac{\Delta {\text{H}}^{\circ }-\text{T}\Delta S^{\circ }}{-\text{RT}}$$

The *enthalpic contribution* to complex stabilization derives from the metal ion preference for particular donors. Such a preference was described early on in a concept that grouped metal ions into two categories, A and B [38]. The grouping depends on whether the complex is more stable with a donor atom from a member of the first periodic group (F, O, N) or with a member of a subsequent group (Cl, S, P). In this classification, Zn(II) belongs into category B, in which neither the charge nor the size of the interacting atoms/ions is the primary reason for the stability. Stability of the complex increases with the ionization potential of the metal (charge density) and with decreasing electronegativity of the ligand donors, ie. the tendency of a ligand to donate electrons. In the series F, O, N, Cl, Br, I, and S, the electronegativity decreases from left to right while the stability of the complexes with zinc increases. In another concept, metal ions and ligands are grouped into soft and hard acids and bases (SHAB) depending on the degree of their polarizability [39]. In this classification, Zn(II) belongs to a borderline category of acids with a tendency to form stable complexes with moderately polarizable ligands (bases) such as nitrogen donors. The borderline character of Zn(II) is also seen in its tendency to form complexes with both hard oxygen and soft sulfur donors in biological complexes with low molecular weight (LMW) or high molecular weight (HMW) ligands. Zinc forms stable octahedral complexes with l-histidine and citrate with oxygen and nitrogen donors [40], [41] as well as interacts with up to four sulfur donors from cysteine residues in tetrahedral zinc-thiolate sites in proteins, where binding can include nitrogen donors from histidine residues [6], [42]. Enthalpic effects on complex stability are frequently hidden deep in the protein structure. Additional interactions such as hydrogen bonding and electrostatic interactions within the ligand formed during the complexation also affect the stability significantly [3], [5], [24], [43].

The *entropic contribution* to complex stabilization derives mostly from the chelate effect mentioned above and the structure of the protein or LMW ligand when it changes during complexation. The chelate effect is particularly prominent in proteins when the zinc binding site is not preformed. It can be readily seen in the complexation of zinc with EDTA when six water molecules are replaced by one ligand molecule of EDTA (Eq. (18)).

(18) $${\left[\text{Zn}{\left({\text{H}}_{2}\text{O}\right)}_{6}\right]}^{2+}+{\text{EDTA}}^{2-}⇄{\text{ZnEDTA}}^{2-}+4{\text{H}}_{2}\text{O}+2{\text{H}}_{3}{\text{O}}^{+}$$

The increase in the number of ligands (products) provides an entropic driving force for the reaction. The increased entropy is associated with higher complex stabilization (Eq. (17)). Aside from this chelate effect referring the formation of a ring structure, stability of zinc complexes can increase by a macrochelate effect, which refers to entropic effects in the outer coordination sphere, which is common in proteins.

Important issues for complex formation are the charge of the ligand and its acid-base properties. Most of the biological ligands possess acid-base properties and their complexation is linked to the loss of proton(s) (Eqs.(19), (20), (21)).

(19) $$\text{RSH}+{\text{M}}^{n+}⇄\text{RS}-{\text{M}}^{\left(n-1\right)+}+{\text{H}}^{+}$$

(20) $${\text{RNH}}_{3}^{+}+{\text{M}}^{n+}⇄{\text{RNH}}_{2}-{\text{M}}^{n+}+{\text{H}}^{+}$$

(21) $$\text{R}\left(\text{COOH}\right){\text{NH}}_{3}^{+}+{\text{M}}^{n+}⇄\text{R}\left(\text{COO}\right){\text{NH}}_{2^{-}}{\text{M}}^{\left(n-1\right)+}+2{\text{H}}^{+}$$

The strength of competition between metal ions and protons is strictly related to the complex stability and the acid-base properties of the ligand. In general, stabilities of complexes are higher at higher pH because the competition between metal ions and protons decreases. The association or formation constant (*K* or *K*_a_, unit M^−1^) or the dissociation constant (*K*_d_, unit M) describe the stability of the complex at particular conditions (temperature and ionic strength, *I*). If the constants describe the same equilibrium their values are reciprocals: *K*_a_ = 1/*K*_d_. If complexation is associated with the dissociation of protons, calculation of *K*_a_ or *K*_d_ requires knowledge of the acid dissociation constants of the ligand donors.

Most of the biologically important metal complexation equilibria occur at a constant pH value, ie. they are pH-buffered. In this case, the association constant is defined in a much simpler way (Eqs. (22), (23))). In order to differentiate between values that refer to unbuffered and buffered conditions the latter values are called apparent (*K*^app^ or conditional constants referring only to a particular pH value). Determination of apparent constants does not require knowledge of the acid-base properties of the ligand (L), which is a convenient way of describing binding properties of macromolecules.

(22) $${\text{L}}^{x}+{\text{M}}^{n+}⇄{\text{ML}}^{x+n}$$

(23) $$K_{a}^{app}=\frac{\left[{\text{ML}}^{x+n}\right]}{\left[{\text{L}}^{x}\right]\left[M^{n+}\right]}$$

**Fig. 5:**
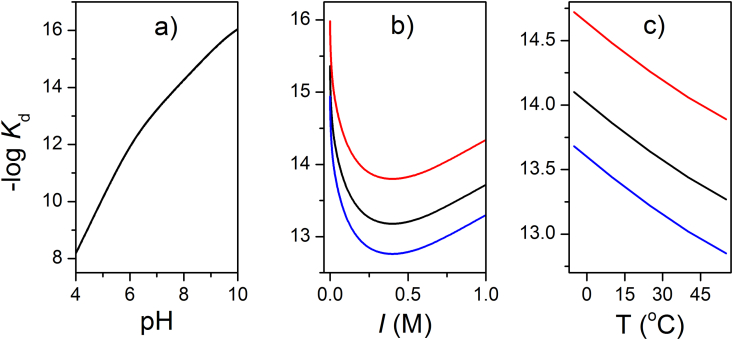
The dependence of the dissociation constant of the [Zn(EDTA)]^2-^ complex on experimental conditions used for its determination such as pH (a), ionic strength, *I* (b) and temperature (c). Red, black and blue colors in b) and c) correspond to pH 7.0, 7.4 and 8.0, respectively.

With the known values of the pH-dependent association constant and the acid dissociation constants one can calculate the apparent constant at any pH value. This conversion is very important in biology. For example, for the formation of the Zn-EDTA complex (*K*_a_ = 16.46), one calculates *K*_a_^app^ = 13.22 and 13.64 at pH 7.0 and 7.4, respectively (Fig. 5a) [44]. Ionic strength and temperature also have a significant influence on the stability of the complex (Fig. 5b,c). The conversion of *K*_a_ to *K*_a_^app^ is important when using tabulated data of *K*_a_, for example for zinc chelators (Table 3). Their binding constants need to be converted to the proper conditions of the experiment (pH, ionic strength and temperature).

In order to determine apparent stability constants of the agents that potentially interact with Zn^2+^ one needs a readout such as a change of absorbance, fluorescence, ellipticity etc. upon Zn^2+^ binding. The constants can be determined in direct titration experiments if the affinity of Zn^2+^ towards the molecule of interest is not lower than ∼10^−8^ M. This value depends on the physicochemical property investigated under Zn^2+^ binding and the ratio between the maximal and minimal value of the measured parameter, i.e. the sensitivity, and the concentrations of reactants. Indirect determinations can be performed in zinc-buffered media. In both applications, knowledge about the stoichiometry of the interaction is required as it affects the numeric value and unit of the constant. Such information can be obtained by Job's method of continuous variation [45]. Metal buffers are solutions of chelating agents with various amounts of total zinc and they are chosen for buffering in certain pZn ranges (Table 3). Ideally they form 1:1 complexes. Control of pZn values from the low attomolar range in the case of TPEN to micromolar in the case of IDA is possible. The values of pZn are calculated by Eq. (3) and depend on pH, temperature and ionic strength. The calculation can be performed using any of the available programs, such as the simplest, MaxChelator, or more advanced ones such as MINEQL+ and Hyperquad Simulation and Speciation software [46], [47], [48], [49]. All mentioned programs contain sets of stability data for various metal ions under various conditions. The more advanced software allows for developing models based on a particular chelator or the stability determined at various conditions. The most common format available in those software packages are conditional (not apparent) pH-independent constants that are tabulated from several databases such as those from IUPAC, NIST, or the Martell and Smith compendium [44]. Results are usually presented as pZn as a function of the investigated physical parameter and are fitted to single or multiple binding models using software such as Origin (OriginLab Corporation), SigmaPlot (Systat Software Corporation), DynaFit (BioKin Ltd). Frequently, data are fitted to Hill's equation that allows for evaluation of whether or not the process involves cooperativity in binding [50]. Two conditions must be fulfilled: the reactions need to reach equilibrium and the interference of the metal buffer with the reaction must be negligible. If it is not negligible calculation must include the distribution between the metal buffer and the ligand under investigation.

Calculations with stability constants become more complexes for stoichiometries other than 1:1 between metal ion and ligand. For instance, most amino acids form ZnL, ZnL_2_, or even ZnL_3_ complexes with zinc ions. Formation of each complex is defined by a separate equilibrium, leading stepwise to the complexes, ie. ZnL_2_ is formed by association of a ligand molecule to the ZnL species (*K*_2_) and ZnL_3_ is formed from ZnL_2_ (*K*_3_). The formation of ZnL_2_ also can be described by the cumulative formation constant β(ZnL_2_) which is *K*_1_ × *K*_2_. Frequently, β values are given as association and dissociation constants but then their units differ from those of M^−1^ and M. Thus, dissociation of the ZnL_2_ complex to Zn^2+^ and two molecules of L has the unit M^2^. The pH-dependent, stepwise or cumulative association constants also require knowledge of the acid-base properties of the ligand in order to calculate the apparent values of ZnL, ZnL_2_ and ZnL_3_ complexes (*K*_1_^app^, *K*_2_^app^, *K*_3_^app^ or β(ZnL)^app^, β(ZnL_2_)^app^ and β(ZnL_3_)^app^). All these equilibria, which differ for ligands, cause a significant problem for comparison of zinc affinities of ligands that form complexes with various stoichiometries. A straightforward comparison of binding ability can be performed only for apparent constants of complexes with the same stoichiometry. In order to be able to compare complexes with different stoichiometries, the concept of a competitivity index has been introduced [40]. Accordingly, all zinc complexes at particular conditions with various stoichiometries and protonation states are summed up and treated as a complex of a virtual molecule Z (Eq. (24)). The Competitivity Index (*CI*) is then the dissociation constant value of the MZ species defined by Eq. (25).

(24) $$\left[\text{MZ}\right]=\sum_{ijk}\left[{\text{M}}_{i}{\text{H}}_{j}{\text{L}}_{k}\right]$$

(25) $$CI=\frac{\left[\text{M}\text{Z}\right]}{\left[\text{M}\right]\left[\text{Z}\right]}$$

This concept, with all its limitations, allows comparison of simple ligands with much more complicated ones in terms of their protonation and stoichiometry at a particular pH value. *CI* indices are included in Table 2 and provide a convenient means of comparing zinc binding ability of the number of pH buffer components, disulfide reductants, carboxylic acids, amino acids, nucleotides, natural redox buffers, antibiotics, etc.

The pH dependence of complexation has important implications for biology because there are significant differences of pH values in different organs and even in different cellular compartments. Increasing the pH value causes deprotonation of a ligand with acid-base properties and complexation while decreasing the pH has the opposite effect. The acid-base properties of the ligand change with complexation: strong binding perturbs the p*K*_a_ considerably while weaker binding has a smaller influence on the p*K*_a_ value of the ligand.

## 5 The coordination of zinc in the “free” zinc pool

While the first part of this article addressed the chemical principles of zinc coordination (water, anions, and other ligands) as relevant for biology, the second part amplifies the subject of biological significance, in particular (i) the speciation of zinc ions *in vivo* and the few instances where LMW ligands interacting with zinc have been identified for specific functions and (ii) the coordination chemistry of the LMW “tools” employed for investigating zinc ions in biology.

**Fig. 6:**
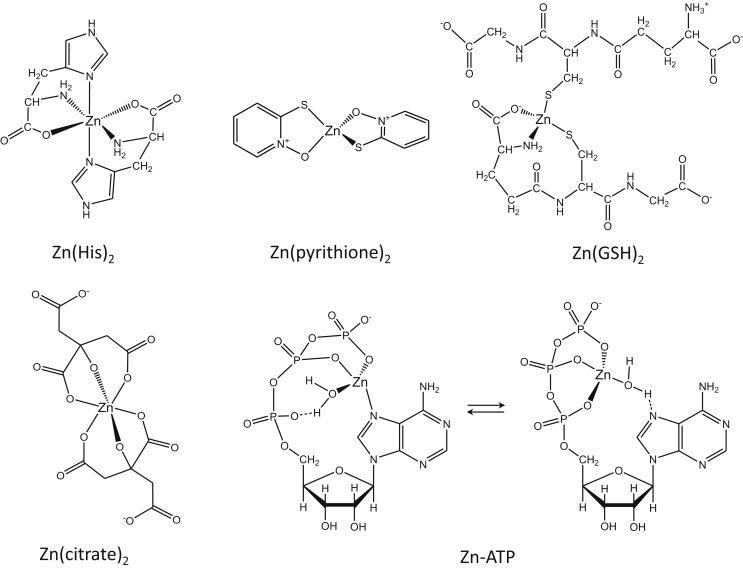
Examples of structures of zinc complexes with low molecular weight biological ligands.

Ligands of the pool of cellular “free” zinc ions (not protein-bound) and the ligands of zinc ions during transport or transfer are generally not considered and knowledge is very limited though some investigations indicate a role of specific complexes. It is a fundamentally important issue which zinc species proteins recognize, and it may be a key to specificity, but a lack of methods to investigate speciation at the very low concentrations of zinc ions has precluded progress. However, there are at least two reports where extracellular interactions of specific species of zinc with proteins have been investigated. For example, it has been demonstrated that a particular species of zinc(II) is involved in metal association with a protein. The species Zn(His)^+^ was found to be the preferred substrate for membrane transport, suggesting a role of amino acids in biological zinc complexation [51]. Histidine forms 1:1 and 2:1 complexes with zinc (Table 2, Fig. 6). The 2:1 complex Zn(His)_2_ has no overall charge. The species relevant for inhibition of carboxypeptidase A was identified as Zn(OH)^+^ [19]. In importance of zinc speciation and overall charge of the zinc complex has been demonstrated by an investigation into the efficacy of zinc lozenges to treat the common cold. Neutral zinc complexes had no effect, negatively charged zinc complexes worsened the outcome, and only positively charged zinc complexes were efficacious [52]. It has been discussed that the rates of zinc transfer would be low on a biologically meaningful time scale at the very low concentrations of zinc ions prevailing in the cytosol. For instance, if concentrations were as low as 1 pM, zinc dissociation rates would be in the order of 14 h, assuming diffusion-controlled association rates [53]. At least two pathways are envisioned to overcome this dilemma. One is direct associative transfer between proteins as has been observed for transfer of zinc between metallothioneins and for transfer or other metal ions by metallochaperones, and the other is a role of LMW compounds in “catalyzing” zinc transfer. Such an enhancement of zinc transfer has been observed in model reactions where compounds that have affinities between those of the zinc donor and the zinc acceptor facilitate metal transfer [54], [55]. Evidence for such reactions being biologically relevant came from the observation that carbonic anhydrase, which exchanges zinc very slowly (t_1/2_ > 100 days), equilibrates rapidly in the cell [56].

Glutathione, ATP, citrate, and amino acids are all candidates for chelating ligands in the LMW pool of zinc (Fig. 6). Evidence for involvement of ATP as a ligand of zinc has been provided, thus demonstrating in principle how biological specificity by LMW species can be achieved. Some kinases phosphorylating vitamins prefer a ZnATP complex over a MgATP complex [57]. In the crystal structures of flavokinase and pyridoxal kinase a bound ZnATP complex was detected [58], [59]. ATP forms a rather stable complex with zinc because the N7 of the adenine moiety provides an additional ligand (*K*_d_ and *CI* value of the ZnL complex is 7.68 × 10^−6^ M; Table 2, Fig. 6) [60], [61].

Glutathione (GSH) as a ligand is particularly intriguing for conferring specificity in metal transfer reactions as zinc forms a variety of complexes with GSH (*K*_d_ of 8.74 × 10^−9^ M^2^ for the ZnL_2_ complex corresponding to a −log*CI* value of 5.16, Table 2, Fig. 6) as a function of pH and GSH concentrations, including ternary complexes [40], [62], [63]. GSH has been discussed as a ligand of the LMW Fe(II) pool [64].

Further exploration of whether or not complexation or changes in complexation of zinc ions determines specificity in biological processes is an important issue for understanding how zinc is provided to proteins for binding, how cellular sensors and transporters recognize zinc, and for how and where zinc expresses its function as a signalling ion at target sites in proteins [65]. In the absence of knowledge of the coordination of zinc in the LMW zinc pool, virtually all experiments employ zinc salts in biological buffers without recognizing that the species of zinc in such solutions most likely are not the biological substrates.

One important aspect of the homeostatic control and re-distribution of zinc in humans is its storage in cellular vesicles, which are solute compartments with properties different from the cytosol. In most instances, it is not known in which chemical form zinc is stored in intracellular vesicles, and how its coordination changes once it is released from the vesicles. Vesicular exocytosis releases zinc into the extracellular fluid, and brings zinc into a biological compartment that provides different coordination environments. In zinc-rich neurons in the brain, zinc is co-released with glutamate from vesicles that are loaded with zinc by the transporter ZnT3. Whether zinc forms a complex with glutamate in the vesicles is not known. The *K*_d_^app^ of the ZnL_2_ complex with glutamate is 2.18 × 10^−6^ M^2^, with a −log*CI* value of 2.7 corresponding to the strength of the interaction of zinc with HPO_4_^2−^ (Table 2) [66]. Preliminary investigations of zinc-rich vesicles by extended X-ray absorption fine structure (EXAFS) spectroscopy demonstrated O, N, and S-coordination [67]. Presumably, at the lower pH value in the vesicles zinc ions are more weakly bound. Upon exocytosis zinc is released into the synaptic cleft, which has a higher pH, and thus it is expected to be bound more tightly. In the endocrine pancreas, zinc is transported into insulin-containing secretory granules of β-cells by the transporter ZnT8, where it has a role in storing crystalline insulin as a hexamer with two bound zinc ions. The coordination environment of zinc depends on whether the hexamer is in the R or in the T state. Zinc is co-secreted with insulin (and amylin), and monomeric insulin coordinates zinc with a *K*_d_ of 0.4 μM [68]. In the exocrine pancreas and in epithelia of mammary glands, the transporter ZnT2 loads the vesicles with zinc. In the exocrine pancreas, zinc it thought to have a role in inhibiting proteases stored in these vesicles. In the mammary glands, vesicular zinc is exocytosed into the milk. In addition to being bound to proteins, zinc is bound in a LMW pool in milk, which has been identified as a zinc-citrate complex in humans [69], [70]. Zinc ions form ZnL and ZnL_2_ complexes with citrate with different overall charges. The ZnL_2_ complex is favored because its *K*_d_ is 1.17 × 10^−12^ M^2^ corresponding to a −log*CI* value of 8.93 (Table 2, Fig. 6) [41]. Micromolar concentrations of picolinate were also found in human milk and a zinc-picolinate complex was isolated [71]. The prostate is another organ where zinc is secreted from vesicles into the prostate fluid. In the prostate, zinc inhibition of aconitase is thought to be responsible for the accumulation of citrate, which could serve as a zinc ligand in prostatic secretion and in seminal fluid [72]. The coordination environment of zinc in other zinc-storing vesicles and its change when secreted from a variety of other zinc-secreting cells is not known, nor is it known in which form cytosolic zinc is provided to the ZnT transporters. The LMW ligands of zinc in urine, blood or other biological fluids have not been identified.

It is not known to which extent LMW ligands participate in zinc buffering, whether zinc buffering occurs exclusively by proteins, LMW complexes, or a combination of both, and to which extent the buffering capacity changes and the pZn differs in different cell types.

Bacillithiol has been identified as an important component of zinc buffering in some bacteria [73]. It forms a ZnL_2_ complex with an apparent *K*_d_ of 5.38 × 10^−13^ M^2^ (−log*CI* = 9.05). Like glutathione it is a redox active component of the LMW zinc pool, linking the availability of zinc from this pool to the redox state of the cell. Thus, not only is the protein pool with zinc coordination to sulfur redox-sensitive, but also the LMW pool.

There is evidence for the participation of *metallochaperones* and *metallophores* in the acquisition and redistribution of zinc. The nomenclature is not consistent. Metallochaperones are proteins. Based on the definition that metallochaperones metallate apoproteins to make metalloproteins metallothioneins classify as zinc chaperones. However, the term has also been used for chemically synthesized LMW compounds that can deliver zinc from the blood to cellular proteins owing to (i) affinities between those of blood proteins such as albumin and cellular zinc metalloproteins and (ii) being sufficiently hydrophobic to cross biological membranes, i.e. they are ionophores [74]. Metallochaperones for zinc have been identified in the periplasmic space of bacteria [75]. It is a pressing issue whether or not, and if so under which circumstances, metallochaperones for zinc are required in eukaryotes. Employing zinc chaperones may not to be an obligatory process in zinc re-distribution in eukarya as too many chaperones would be required to provide specificity for interaction with the about 3000 predicted human zinc proteins.

Zincophores similar to the established siderophores (Fe) and chalcophores (Cu) have been described. Zincophores are different from ionophores that transport zinc through the membrane, such as pyrithione which forms a neutral ZnL_2_ complex (Fig. 6) with an apparent *K*_d_ of 5.0 × 10^−12^ M^2^ (−log*CI* = 8.30, Table 2) [76]. Zincophores are typically found in bacteria that need to acquire Zn^2+^ in an environment with either low zinc concentrations or high competition for zinc. Hence, they need relatively high affinity to scavenge zinc ions. One zincophore is ethylene-*N,N*′-diaminedisuccinic acid ([S,S]-EDDS) [77], which has a *K*_d_ of 2.3 × 10^−11^ M (−log*CI* = 10.6, Table 3). Its high affinity for zinc is comparable with common chelators such as EGTA and HEDTA [44]. Evidence is now accumulating that some siderophores also may actually be zincophores [78]. The term zincophore has been employed for the protein Pra1 secreted from *C. albicans* and involved in scavenging Zn^2+^ [79]. Pra1 has orthologues in other fungi but not in vertebrates. The complexation chemistry of zincophores is important because they compete with the host for zinc. Therefore, it may be possible to develop new therapeutic agents with antibacterial/antifungal activities.

It is known in the scientific community but not widely acknowledged for practical purposes that several antibiotics bind zinc, e.g. tetracycline and gentamicin (Table 2). When antibiotics are prescribed to treat infections or given prophylactically, the interaction with zinc may be an issue for their efficacy and/or affecting zinc availability and thus the nutritional zinc status of the host. In fact, the importance of this mechanism of action has been demonstrated for vancomycin, which restricts the access of bacteria to zinc [80].

Nicotianamine, an amino acid derivative and a precursor of siderophores, is involved in zinc (and iron) metabolism in plants [81]. Its apparent *K*_d_ is 1.6 × 10^−11^ M (Table 2) [82]. It transports zinc from roots to shoots and is also secreted from the roots in metal hyperaccumulator plants [83]. In this case, instead of being a chelating agent for uptake of a metal, it is secreted to serve as a chelating agent for the excess zinc in order to avoid further uptake. This principle is related to that of other compounds that are antagonists of zinc uptake such as phytate (inositol hexaphosphate). Its *K*_d_ is 3.7 × 10^−11^ M (Table 3) [35].

## 6 The coordination chemistry of “zinc tools”

The “tools” for investigating zinc in biology include at least three types of agents: (i) chelating agents to remove zinc ions or buffer zinc in certain ranges of pZn, (ii) pharmacological agents such as ionophores to transport zinc through cellular membranes and deliver zinc in a bioavailable form, and (iii) chromophoric and fluorescent probes to investigate zinc-dependent processes (Table 3).

The strategy of designing zinc probes is well established and the properties of the probes such as sensitivity, selectivity and affinity have been discussed widely [84]. One major principle is that a chelating agent is coupled to a fluorophore and once zinc is bound, fluorescence is “turned on.” The tools were developed without prior knowledge of the properties of the biological pools of zinc and accordingly a lot of discussions ensued which species they actually measure. For example, probes and sensors purported to measure “free” zinc ions can compete with protein-bound zinc and/or their response can be modified by binding other ligands [65], [85]. For future investigations, it is important that knowledge of zinc speciation informs the development and application of tools.

Two compounds are widely used in zinc biology: 2-mercaptopyridine-N-oxide (pyrithione) (Fig. 6), which is an ionophore and employed to increase cellular zinc ion concentrations, and N,N,N′,N′-tetrakis(2-dipyridylmethyl)ethylenediamine (TPEN), which is a membrane-permeable chelating agent that is employed to bind cellular zinc in order to make it unavailable for other processes (*K*_d_ at pH 7.4 is 6.4 × 10^−16^ M for the ZnL complex, Table 3) [44]. Both agents are used to calibrate cellular zinc probes and sensors by saturating their binding sites (with pyrithione) and by removing their zinc (with TPEN).

Chelating agents are also used as zinc buffers in experimental investigations. Chelating agents that can be employed for buffering zinc over a wide range of pZn values (4.2–16.2) are available and will be an essential tool for future investigations (Table 3).

There are two common issues with zinc complexation when using biological buffers. One is that many pH buffers serve as ligands of zinc. Phosphate buffer forms ZnHPO_4_ (Table 2) and then Zn(OH)_2(s)_ or insoluble zinc phosphates depending on the actual pH of the buffer and zinc concentrations. Another frequently used buffer, Tris, also forms a weak ZnL complex (*K*_d_ = 33 mM, Table 2). Many Good's buffers have nitrogen donors and bind zinc [86]. HEPES has a sulfonate group that serves as an oxygen donor. Glycine binds zinc with millimolar affinity at neutral pH and much stronger (micromolar affinity) at pH 9, the region in which it is typically employed as a pH buffer (Table 2) [87]. Bis-tris, bis-tris propane, bicine and tricine bind zinc much more efficiently than Tris. The *K*_d_ values for bicine and tricine are ∼10^−5^ M at pH 7.4 (Table 2) [88], [89]. Thus in any zinc competition experiment with moderately strong zinc-binding ligands, the zinc binding capacity of pH buffers needs to be considered. The second issue is the use of reducing agents that are necessary at relatively high concentrations to provide a reducing environment similar to the one in cells. The reducing agent, especially if it is based on thiols, may bind zinc tightly and thus remove zinc from the protein. Also, zinc binding changes the redox properties of the reducing agent. Dithiothreitol (DTT, Cleland's reagent) is widely used for many standard biological buffers. It forms several complexes with zinc with various stoichiometries and with coordination involving its two thiolates. Its affinity towards zinc measured by the *CI* index is nanomolar (−log*CI* = 8.3, Table 2) [90]. A recently introduced reagent dithiobutylamine (DTBA), similar in structure to DTT, binds Zn(II) even more tightly with subnanomolar affinity (−log*CI* = 9.3, Table 2) [91]. Two other reductants frequently used bind zinc less tightly with submillimolar affinity: 2-mercaptoethanol and tris carboxyethyl phosphine (TCEP), which has a more negative redox potential [92], [93].

## 7 Conclusions

Zinc-ligand bonds involve the spherical 4s electron shell and have about 20% covalent character [94]. A consequence is that the zinc ion is stereochemically inactive and quite flexible regarding coordination numbers with coordination dynamics that do not underlie the stereochemical constraints typical for the transition metal ions. It is also different from magnesium (Mg^2+^) and calcium (Ca^2+^) as it forms much stronger complexes with water and a variety of anions and ligands. These characteristics are important for its biological functions. The simultaneous presence of other metal ions and anions in the biological environment sets constraints on the stability of zinc/protein complexes. Zinc exchange kinetics can be restricted in proteins, but the generally fast ligand exchange kinetics endow zinc with characteristics well suited for signalling and re-distribution on biological time scales. The different zinc complexes discussed here are important in zinc transfer between the different pools. Minimally, these pools include equilibria between several species (Eq. (26)).

$${\text{Zn}}_{\left(\text{aq}\right)}^{2+}\;\text{or}\;\text{Zn}-\text{anion}\left(\text{s}\right)⇄\text{Zn}-\text{LMW}\;\text{chelates}\;\left(⇄\text{Zn}-\text{Chaperone}\right)⇄\text{Zn}-\text{Protein}$$

Changes in zinc species are important when zinc is transported through membrane proteins. Hydration chemistry and the ionization of its bound water are important for its function in enzymes (Zn-OH as a nucleophile), where it also forms complexes with anions and chelating ligands. Furthermore, knowledge of zinc species informs the formulation of zinc complexes for use in animal feed, where often ZnO is used, for bioavailability when supplemented in the human diet [95], when performing experiments with cultured cells to increase cellular zinc, to treat a zinc deficiency, or when chelating agents are employed to decrease zinc concentrations. Knowledge of zinc chemistry impacts many areas, e.g. nutrition, toxicology, and pharmacology. It is anticipated that different zinc species provide biological specificity. Finally, it is a pressing issue to determine zinc species to understand transporter characteristics, zinc sensing, and zinc signalling. In the absence of knowledge about LMW ligands, virtually all experiments are performed with zinc salts and thus inappropriate substrates for biological interactions.

## Figures and Tables

**Table 1:** Concentrations of simple biological and inorganic anions and stepwise formation constants for zinc anion complexes. Data refer to 25 °C and *I* = 0.1 M unless otherwise specified.

Anion	Cell (mM)	Blood (mM)	Zn(II) complex formation	log*K*	Reference
ClO_4_^−^	*n.d.*	*n.d.*	[Zn(H_2_O)_*x*_]^2+^ + ClO_4_^−^ ⇆ [Zn(ClO_4_)(H_2_O)_*x-1*_]^+^	−1.58	[96]
NO_3_^−^	*n.d.*	*n.d.*	[Zn(H_2_O)_*x*_]^2+^ + NO_3_^−^ ⇆ [Zn(NO_3_)(H_2_O)_*x-1*_]^+^	−0.12	[97]
Cl^−^	4	116	[Zn(H_2_O)_*x*_]^2+^ + Cl^−^ ⇆ [ZnCl(H_2_O)_*x-1*_]^+^ [ZnCl(H_2_O)_*x-1*_]^+^ + Cl^−^ ⇆ [ZnCl_2_(H_2_O)_*x-2*_] [ZnCl_2_(H_2_O)_*x-2*_] + Cl^−^ ⇆ [ZnCl_3_(H_2_O)_*x-3*_]^−^ [ZnCl_3_(H_2_O)_*x-3*_]^−^ + Cl^−^ ⇆ [ZnCl_4_]^2-^	0.43^a^ 0.18^a^ −0.1^a^ −0.31^a^	[98], [99]
HCO_3_^−^	12	29	[Zn(H_2_O)_*x*_]^2+^ + HCO_3_^−^ ⇆ [Zn(HCO_3_)(H_2_O)_*x-1*_]^+^ [Zn(H_2_O)_*x-1*_]^2+^ + CO_3_^2−^ ⇆ [Zn(CO_3_)(H_2_O)_*x-1*_]_(aq)_	0.85^b^ 3.3^a^	[98], [100]
OAc^−^	*n.d.*	*n.d.*	[Zn(H_2_O)_*x*_]^2+^ + OAc^−^ ⇆ [Zn(OAc)(H_2_O)_*x-1*_]^+^	0.93	[101]
SO_4_^2-^	0.3	0.27 (serum)	[Zn(H_2_O)_*x*_]^2+^ + SO_4_^2−^ ⇆ [Zn(SO_4_)(H_2_O)_*x-1*_]	2.34^c^	[102], [103], [104]
P_i_ (p*K*_a_ = 7.21)^d^	0.5–5	0.8–1.5	[Zn(H_2_O)_*x*_]^2+^ + HPO_4_^2−^ ⇆ [Zn(HPO_4_)(H_2_O)_*x-1*_]_(aq)_	2.4	[95], [96], [97], [98], [99], [100], [101], [102], [103], [104], [105], [106], [107]
HS^−^	15 nM	*n.d.*	[Zn(H_2_O)_*x*_]^2+^ + HS^−^ ⇆ [Zn(HS)(H_2_O)_*x-1*_]^+^ [Zn(HS)(H_2_O)_*x-1*_]^+^ + HS^−^ ⇆ [Zn(HS)_2_(H_2_O)_*x-2*_]_(aq)_	6.1^e^ 4.1^e^	[108], [109]
HO^−^	0.1 μM	*n.d.*	[Zn(H_2_O)_*x*_]^2+^ + OH^−^ ⇆ [Zn(OH)(H_2_O)_*x-1*_]^+^ [Zn(OH)(H_2_O)_*x-1*_]^+^ + OH^−^ ⇆ [Zn(OH)_2_(H_2_O)_*x-2*_]_(aq)_ [Zn(OH)_2_(H_2_O)_*x-2*_]_(aq)_ + OH^−^ ⇆ [Zn(OH)_3_(H_2_O)_*x-3*_]^-^ [Zn(OH)_3_(H_2_O)_*x-3*_]^2+^ + OH^−^ ⇆ [Zn(OH)_4_]^2-^	4.95^f^ 4.25^f^ 3.9^f^ 3.5^f^	[22]

*n.d.* – not determined.

a *I* = 0 M.

b *I* = 0.68 M.

c *I* is unknown.

d P_i_ refers to inorganic phosphate, which in water medium is H_2_PO_4_^−^ in equilibrium with HPO_4_^2−^, p*K*_a_ corresponding to H_2_PO_4_^−^ dissociation is 7.21 [107].

e *I* = 0.72 M.

f Constants determined in water from measurements by atomic absorption spectrophotometry.

**Table 2:** The apparent dissociation constants and competitivity indices (*CI*)^a^ of zinc complexes with common chemicals and natural products. Unless otherwise specified, the stability values were determined at pH 7.4, *I* = 0.1 M and 25 °C.

Ligands	Zinc ligand	Complex stoichiometry^b^	Donors c	Apparent dissociation constant (*K*_d_)	p*K*_d_	Competitivity Index (−log*CI*)^a^	References
pH buffers	Tris	ZnL	NO^∗^	3.29 × 10^−2^ M	1.48	1.48	[110]
	Bis-Tris	ZnL	NO^∗^	5.03 × 10^−3^ M	2.30	2.30	[111]
	Bis-Tris propane	ZnL	NO^∗^	1.19 × 10^−4^ M	2.93	3.32	[112]
		ZnL_2_	N_2_O_2_^∗^	2.15 × 10^−6^ M^2^	5.66		
	Bicine	ZnL	NO^∗^	7.50 × 10^−5^ M	4.12	4.39	[88]
		ZnL_2_	N_2_O_2_^∗^	3.12 × 10^−7^ M^2^	6.51		
	Tricine	ZnL	NO^∗^	6.47 × 10^−5^ M	4.19	4.19	[89]
Reductants	TCEP	ZnL	O_3_P	3.29 × 10^−3^ M	3.29	3.29	[93]
	β-Mercaptoethanol	Zn_2_L_3_-Zn_6_L_15_	S_2_–S_4_ (S_4_)	*n.c.*	*n.c.*	3.60^d^	[92]
	DTT	ZnL_2_, Zn_3_L_4_ (ZnL)	S_4_ (S_4_)	2.83 × 10^−10^ M^2^	9.55	8.32	[90]
	DTBA	ZnL_2_ (ZnL_,_ Zn_2_L_3_)	S_4_ (S_4_)	5.38 × 10^−13^ M^2^	12.27	9.29	[91]
Carboxylic acids	α-Ketoglutaric acid	ZnL, ZnL_2_	O_2_, O_4_	∼2 × 10^−2^ M^2^	∼1.7	1.13^d^	[113]
	Pyruvic acid (pyruvate)	ZnL, ZnL_2_	O_2_, O_4_	∼2 × 10^−3^ M^2^	∼2.7	1.26^d^	[114]
	Succinic acid (succinate)	ZnL	O_2_	2.54 × 10^−2^ M	1.60	1.60	[115]
	Glutaric acid (glutarate)	ZnL	O_2_	2.52 × 10^−2^ M	1.60	1.60	[115]
	Lactic acid (lactate)	ZnL	O_2_	1.38 × 10^−2^ M	1.86	1.86	[116]
	Lipoic acid^e^	ZnL	O_2_	3.7 × 10^−3^ M	2.43	2.43	[117]
	Malic acid (malate)	ZnL	O_2_	1.52 × 10^−4^ M	3.82	3.82^f^	[118]
	Folic acid (folate)	ZnL_2_	N_2_O_2_	2.2 × 10^−6^ M^2^	5.67	2.84^g^	[119]
	Oxalic acid (oxalate)	ZnL	O_2_	3.16 × 10^−4^ M	3.50	3.50	[120]
	Orotic acid (orotate)	ZnL	NO	2.20 × 10^−4^ M	3.66	4.80^f^	[121]
		ZnL_2_	N_2_O_2_	2.40 × 10^−8^ M^2^	7.62		
	Dipicolinic acid	ZnL	NO_2_	5.74 × 10^−4^ M	3.24	8.70^h^	[122]
		ZnL_2_	N_2_O_4_	1.33 × 10^−12^ M^2^	11.88		
	Citric acid (citrate)	ZnL_2_ (ZnL)	O_6_ (O_6_)	1.17 × 10^−12^ M^2^	11.93	8.93	[41]
Amino acids	Glutamic acid (glutamate)	ZnL	NO	2.85 × 10^−3^ M	2.54	2.70	[66]
		ZnL_2_	N_2_O_2_	2.18 × 10^−5^ M^2^	4.66		
	Glycine	ZnL	NO	2.40 × 10^−3^ M	2.62	2.82	[87]
		ZnL_2_	N_2_O_2_	1.29 × 10^−5^ M^2^	4.89		
	Aspartic acid (aspartate)	ZnL	NO	3.92 × 10^−4^ M	3.40	3.54	[44]
		ZnL_2_	N_2_O_2_	4.13 × 10^−6^ M^2^	5.38		
	N-Ac-Cys	ZnL_2_ (ZnL)	O_2_S_2_ (OS)	6.77 × 10^−8^ M^2^	7.17	4.06	[123]
	Histidine	ZnL	N_2_O	3.22 × 10^−4^ M	3.49	5.48	[40]
		ZnL_2_	N_4_O_2_	3.05 × 10^−9^ M^2^	8.52		
	Cysteine	ZnL_2_ (ZnL)	N_2_S_2_ (NS)	9.77 × 10^−12^ M^2^	11.01	7.84^i^	[124]
	d-penicillamine	ZnL_2_ (ZnL)	N_2_S_2_ (NS)	2.81 × 10^−12^ M^2^	11.55	8.38^i^	[124]
Nucleotides	AMP	ZnL	NO	1.84 × 10^−3^ M	2.73	2.73	[125]
	ADP	ZnL	NO_2_	9.73 × 10^−5^ M	4.38	4.38	[125]
	ATP	ZnL	NO_3_	7.68 × 10^−6^ M	5.11	5.11	[125]
Redox buffers	GSSG	ZnL (Zn_2_L)	N_2_O_2_ (NO)	5.3 × 10^−5^ M	4.28	4.28	[66]
	GSH	ZnL	NOS	1.65 × 10^−4^ M	3.78	5.16	[126]
		ZnL_2_	NOS_2_	8.74 × 10^−9^ M^2^	8.06		
	Bacillithiol	ZnL	NSO_2_^∗^	4.0 × 10^−6^ M	5.40	9.05^j^	[73]
		ZnL_2_	N_2_S_2_^∗^	5.38 × 10^−13^ M^2^	12.27		
Others	Tetracycline	ZnL	O_2_	6.77 × 10^−3^ M	2.17	2.17^f^	[127]
	Carnosine	ZnL	NO	4.55 × 10^−3^ M	2.34	2.34	[128]
	Oxytetracycline	ZnL	O_2_	2.24 × 10^−3^ M	2.65	2.65^f^	[127]
	Diphosphate (pyrophosphate)	ZnL	O_2_	1.91 × 10^−3^ M	2.72	2.72	[32]
	Gentamicin C1a	ZnL	NO^∗^	1.60 × 10^−3^ M	2.79	2.79	[129]
	Histamine	ZnL	N_2_	2.98 × 10^−3^ M	2.53	2.96	[130]
		ZnL_2_	N_4_	5.41 × 10^−6^ M^2^	5.27		
	Triphosphate	ZnL	O_3_	1.35 × 10^−7^ M	6.87	6.87	[33]
	Tetraphosphate	ZnL	O_4_^∗^	5.72 × 10^−8^ M	7.24	7.24	[34]
	Pyrithione	ZnL_2_^k^ (ZnL)	O_2_S_2_^k^ (OS)	5.0 × 10^−12^ M^2^	11.30	8.30	[76]
	Phytic acid (phytate)	ZnL	O_5_^∗^	3.7 × 10^−11^ M	10.44	10.44	[35]
	Nicotianamine	ZnL	N_3_O_3_	1.60 × 10^−11^ M	10.48	10.48	[82], [131]

a *CI* is the apparent dissociation constant of ZnZ complex (zinc complex of theoretical molecule Z), such that [ZnZ] = Σ_ijk_ [Zn*i*H*j*L*k*], at a given overall component concentration. The concentrations of Z were set at 2 mM, and those of Zn^2+^ at 0.5 mM.

b Complexes in brackets are the minor species.

c Donors in brackets refer to complex as minor species.

d *I* = 0.5 M.

e Dihydrolipoic acid (reduced form of lipoic acid) is assumed to bind Zn^2+^ more tightly.

f 37 °C, *I* = 0.15 M.

g 20 °C, *I* = 0.01 M.

h 20 °C.

i *I* = 0.2 M.

j Apparent dissociation constant determined at pH 7.7, *I* = 0.15 M.

k Complex in crystals present as dinuclear Zn_2_L_4_ species with S_3_O_2_ donors. *n.c*. – not calculated. Asterisks denote putative donor atoms.

**Table 3:** The apparent dissociation constants^a^ of zinc complexes with widely used zinc chelators and fluorogenic or chromophoric probes with pZn ranges of their application.^b^ Unless otherwise specified, the stability values were determined at pH 7.4, *I* = 0.1 M and 25 °C. Stoichiometry is simplified to ZnL_*x*_ without protonation state.

	Chelator/probe	Donors	Complex stoichiometry	Apparent dissociation constant (*K*_d_)	−log *K*_d_ (p*K*_d_)	pZn range of the application	References
Zinc chelators	TPEN	N_6_	ZnL	6.4 × 10^−16^ M	15.2	14.2–16.2	[44]
	DTPA	N_2_O_4_	ZnL	5.6 10^−15^ M	14.3	13.3–15.2	[44]
	EDTA	N_2_O_4_	ZnL	2.3 × 10^−14^ M	13.6	12.7–14.6	[44]
	HEDTA	N_2_O_3_	ZnL	6.6 × 10^−13^ M	12.2	11.2–13.1	[44]
	EDDS^c^	N_2_O_4_	ZnL	2.3 × 10^−11^ M	10.6	9.7–11.6	[44]
	BAPTA	N_2_O_4_	ZnL	4.9 × 10^−10^ M	9.3	8.4–10.3	[132]
	EGTA	N_2_O_4_	ZnL	6.3 × 10^−10^ M	9.2	8.2–10.2	[44]
	EDDA	N_2_O_2_	ZnL	1.2 × 10^−9^ M	8.9	8.0–9.9	[44]
	NTA	NO_3_	ZnL	4.4 × 10^−9^ M	8.4	7.4–9.3	[133]
	Cyclam^d^	N_4_	ZnL	2.0 × 10^−9^ M	8.7	7.5–9.5	[134]
	IDA	NO_2_	ZnL	3.2 × 10^−5^ M	4.5	5.3–6.8	[135]
		N_2_O_4_	ZnL_2_	4.8 × 10^−9^ M^2^	8.3		
Fluorescent probes	FluoZin-3	N_2_O_3_	ZnL	8.9 × 10^−9^ M	8.1	7.1–9.0	[9]
	RhodZin-3	N_2_O_3_	ZnL	1.4 × 10^−9^ M	8.9	7.9–9.8	[136]
	ZnAF-1	N_4_	ZnL	7.8 × 10^−10^ M	9.1	8.2–10.1	[137]
	ZnAF-2	N_4_	ZnL	2.7 × 10^−9^ M	8.6	7.6–9.5	[137]
	ZnAF-1F	N_4_	ZnL	2.2 × 10^−9^ M	8.7	7.7–9.6	[137]
	ZnAF-2F	N_4_	ZnL	5.5 × 10^−9^ M	8.3	7.3–9.2	[137]
	Zinpyr-1^e^	N_3_O	ZnL	7 × 10^−10^ M	9.2	8.2–10.1	[138]
	Zinpyr-4^e^	N_4_	ZnL	6.5 × 10^−10^ M	9.2	8.2–10.1	[139]
	NBD-TPEA^f^	N_5_	ZnL	2 × 10^−9^ M	8.7	7.8–9.7	[140]
	Zinbo-5^f^	N_3_O	ZnL	2.2 × 10^−9^ M	8.7	7.7–9.6	[141]
	Fura-2^f^	N_2_O_4_	ZnL	3 × 10^−9^ M	8.5	7.5–9.4	[142]
	Mag-Fura-2	NO_3_	ZnL	2 × 10^−9^ M	8.7	7.7–9.6	[143]
	NewPort Green DCF^g^	N_3_	ZnL	1 × 10^−6^ M	6.0	5.1–6.9	[144]
	NewPort Green PDX^e^	N_3_	ZnL	4 × 10^−5^ M	4.4	3.9–5.4	[145]
	Zinquin^h^	N_4_	ZnL	3.7 × 10^−7^ M	6.4	7.0–9.3	[146]
			ZnL_2_	8.5 × 10^−13^ M^2^	13.1		
Chromophoric probes	Zincon	N_2_O_2_	ZnL	1.3 × 10^−5^ M	4.9	4.2–5.9	[147]
	PAR	N_2_O	ZnL	2.75 × 10^−5^ M	4.6	7.3–9.4	[148]
		N_4_O	ZnL_2_	7.08 × 10^−13^ M^2^	12.2		

a Dissociation constants of ZnL and ZnL_2_ are defined as [Zn^2+^][L]/[ZnL] and [Zn^2+^][L]^2^/[ZnL_2_], respectively.

b The pZn range for application of a particular chelator/probe refers to free zinc values (−log[Zn^2+^]_free_) corresponding to 0.9 mM: 1 mM–0.1 mM: 1 mM molar ratio (Zn^2+^: L) range. For IDA the application range refers to 0.45 mM: 1 mM–0.05 mM: 1 mM molar ratio range. In the case of Zinquin and PAR, the pZn range corresponds to 10 μM: 4.5 μM–10 μM: 0.5 μM and 100 μM: 45 μM–100 μM: 5 μM, respectively due to limited solubility and typical range of use. It should be noted that the range of application for some probes is wider due to a higher signal upon Zn^2+^ binding. For those probes, free zinc below 10% of probe saturation can be measured.

c T = 20 °C.

d *I* = 0.2 M.

e pH = 7.0.

f pH = 7.5.

g Experimental pH value unknown.

h Data determined in HBBS buffer.

## Abbreviations

BAPTA: 1,2-Bis(2-Aminophenoxy)ethane-*N*,*N*,*N*′,*N*′-tetraacetic acid
Bicine: *N,N*-Bis(2-hydroxyethyl)glycine
Bis-Tris: 2,2-Bis(hydroxymethyl)-2,2′,2″-nitrilotriethanol
Bis-Tris propane: 1,3-Bis[tris(hydroxymethyl)methylamino]propane
Cyclam: 1,4,8,11-Tetraazacyclotetradecane
DTPA: *N,N*-Bis(2-[bis(carboxymethyl)amino]ethyl)glycine
EDDA: Ethylenediamine-*N*,*N*′-diacetic acid
EDDS: (*S*,*S*)-Ethylenediamine-*N*,*N*′-disuccinic acid
EDTA: Ethylenedinitrilotetraacetic acid
EGTA: Ethylene glycol-bis(2-aminoethylether)-*N*,*N*,*N*′,*N*′-tetraacetic acid
HEDTA: *N*-(2-Hydroxyethyl)ethylenediamine-*N*,*N*′,*N*′-triacetic acid
IDA: Iminodiacetic acid
NTA: Nitrilotriacetic acid
PAR: 4-(2-Pyridylazo)resorcinol
TPEN: *N*,*N*,*N*′,*N*′-Tetrakis(2-pyridylmethyl)ethylenediamine
Tricine: *N*-[Tris(hydroxymethyl)methyl]glycine
